# Supplementary material for: Effects of interoceptive accuracy in autonomic responses to external stimuli based on cardiac rhythm
Source: PLoS One. 2021 Aug 31;16(8):e0256914. doi: 10.1371/journal.pone.0256914 (PMC8407568; doi:10.1371/journal.pone.0256914)
Supplement: Attachment — Submitted filename: ResponseToReviewersver.docx [file pone.0256914.s003.docx]

We would like to thank the editor and reviewers for their thoughtful comments and advice, which have helped us make appropriate revisions to our manuscript. We also would like to express our deep gratitude to Reviewer 2 for endorsing the publication of our manuscript. Here, we have provided point-by-point responses to these comments and have revised our manuscript accordingly.

To the Associate editor:

*Effect size is still a major concern. Additional works such as statistical cross-validation analyses based on the suggestions from the reviewer may be needed to improve the manuscript further.*

Response: We have taken your concern that the effect size is small seriously and we respect your opinion that we have not secured a sufficient number of participants. Adding more participants was an option to be considered, but laboratory conditions are different now than they were when we conducted this experiment, and it is difficult to add experimental data in the exact same setting. In addition, the COVID-19 pandemic made it extremely difficult to conduct the experiment. We concluded that additional data are not a realistic option for us. On the other hand, the power analysis and bootstrap tests suggested that although it was not high, the value of pη2 was above the minimum size. Therefore, we would like to respond to this comment by adding the following information to the manuscript. We would appreciate your understanding.

[Added text]

Page 5, line 114

A priori power analysis indicated that a sample size of at least 28 was necessary.

Page 10, line 242

(*F* [1.92, 69.10] = 3.32, *p* < .05, *pη*2 = .10, CI - .01 - .20).

Page 14, line 321-322

This study was only able to show minimal effect size. Increasing the research size and increasing the sample size would be necessary to establish this effect as robust.

*Please review your reference list to ensure that it is complete and correct. If you have cited papers that have been retracted, please include the rationale for doing so in the manuscript text, or remove these references and replace them with relevant current references. Any changes to the reference list should be mentioned in the rebuttal letter that accompanies your revised manuscript. If you need to cite a retracted article, indicate the article’s retracted status in the References list and also include a citation and full reference for the retraction notice.*

Response: Thank you for the opportunity to check the references. We reviewed the reference list again and confirmed that no papers had been retracted.

*Reviewer #1:*

*I would like to thank the authors for responding exhaustively to my comments. They also reorganized the manuscript in a way that reads better now.*

*However, although I appreciate the work that they put into the new version of the manuscript and the experimental question that is novel, I still have some methodological issues that cannot be solved at this stage:*

Response: We are grateful for the evaluation of the revised manuscript. We would also like to thank you for your constructive comments and suggestions. We have addressed each point below.

*1) The numbers of trials: There are only 2 trials per condition, and this makes the study very weak.*

Response: Thank you for your comment. This experiment had only two trials for each condition. This is because previous studies included only one trial in the experiments (Iwanaga et al., 2005; Takenaka et al., 2005), and we thought it was better to take as much time as possible for each trial rather than increasing the number of trials. We believe that we could show some effects, even with just two trials. In addition, there was a problem of participants getting fatigued during the experiment. In the present experiment, we alternated the experimental task with a heartbeat detection task or a time estimation task. This method was used to reduce the effects of habituation on continuous stimuli and provide a reliable time for the participants’ heart rates to return to a steady state after the stimuli changed. Therefore, the duration of experiment was longer, with each trial lasting 20-25 min. We believe that increasing the number of trials would further increase the burden on the participants.

[Added text]

Page 14-15, line 336-342

Moreover, there were only two trials in this study, and these were relatively small. Since only a single trial was conducted for each condition in previous studies [21, 43], it was considered more important to take as much time as possible for one trial than to increase the number of trials. In addition, a long experiment may result in increased fatigue of the participants, which may affect their heart rates. Therefore, in the future, increasing the number of trials by dividing the experiment into multiple days may produce results that are more consistent and demonstrate that interoceptive accuracy is related to autonomic reactivity.

*2) The results from the 3-way ANOVA -that I asked to perform in the first revision- show one significant effect that the authors report as p<0.05 and η2 = .003 which is a very small effect size; I wonder if there is an error here. Since the effect size is very small for a significant effect.*

Response: Thank you for raising this concern. We rechecked our statistical calculations and determined that η2 was most likely inappropriate for this experiment. Increasing the sample size is the best way to increase the effect size, but the situation in our laboratory and across the world did not allow for this. We re-calculated *p*η2 and found it to be .10. This further validates the trustworthiness of our experimental results. In addition, the confidence interval for the effect size using the bootstrap method was .01–.20, which we believe increases the reliability of the results.

[Added Text]

Page 10, line 242

(*F* [1.92, 69.10] = 3.32, *p* < .05, *pη*2 = .10, CI - .01 - .20).

*3) The baseline that was not recorded at the beginning of the task.*

Response: Thank you for your essential remarks. As you stated, we did not calculate the speed of the stimulus based on the baseline before the trial. If we assume that the baseline is before the start of the trial, we can calculate the heart rate easily since we measured the participants’ heart rates before the trial. However, there is a possibility that there was an effect from the previous task on participants’ heart rates, and if we calculate the average, although it is supposed to be closer to the actual heart rate than the resting baseline, it is still not synchronized with the actual heart rate. Therefore, we believe that it is best to compare heart rates during stimulus presentation. In addition, our results are based on responses to heartbeat-like stimuli and not on synchronization with actual heartbeats. Therefore, we believe that our results are meaningful even if we did not measure the baseline. While it is tempting to align the heart rates with the baseline, this would be distracting from our goal of examining the relationship between rhythmic sound presentation and heart rate. We hope that you will allow us to finalize this experimental condition.

[Added text]

Page 14-15, line 331 - 336

In addition, the study did not compare the baseline heart rate and the condition in which the stimulus was presented. Although there was a significant difference between these states, the possibility that interoceptive accuracy is involved in switching the state with modality and the state without modality cannot be denied. In future studies, it will be important to increase the inter-trial interval and compare the results with the baseline before the stimulus presentation and between trials.

*My conclusion is that I cannot accept the manuscript in this form. One possibility would be to collect more data, this may compensate for the small numbers of trials. I would recommend them to perform an a-priori power analysis based on literature on similar effects. I think that increasing the number of participants would be informative on the effects that the authors are looking for that seem to have a small effect size for now.*

Response: Thank you for this suggestion. We performed a power analysis, as suggested by the reviewer. We used the traditional effect size of 0.25 because there were no similar results showing effect sizes in previous studies. The results indicated that a reasonable sample size would be 28, which is more than the current number of participants. Due to the miscalculation of the effect size, we are hopeful that this is a reasonable sample size for our results. Increasing the sample size is also very attractive, and we understand that it is an efficient way to increase the effect size. We also considered adding more participants. However, laboratory conditions are different now than they were when we conducted this experiment, and it is difficult to add experimental data in the exact same setting. In addition, the COVID-19 pandemic made it extremely difficult to conduct the experiment. Therefore, we concluded that adding more data is not a realistic option. Since we had an effect size of value of pη2 that was above the minimum threshold, we would like to respond to this comment by adding the following to the manuscript. We would appreciate your understanding.

[Added text]

Page 5, line 114

A priori power analysis indicated that a sample size of at least 28 was necessary.

Page 14, line 321-322

This study was only able to show a minimal effect size. Increasing the research size and increasing the sample size would be necessary to establish this effect as robust.
