# Supplementary material for: Effects of interoceptive accuracy in autonomic responses to external stimuli based on cardiac rhythm
Source: PLoS One. 2021 Aug 31;16(8):e0256914. doi: 10.1371/journal.pone.0256914 (PMC8407568; doi:10.1371/journal.pone.0256914)
Supplement: Attachment — Submitted filename: ResponseToReviewersver.docx [file pone.0256914.s004.docx]

We would like to express our deepest gratitude to the editor and reviewer. The suggestions we have received have helped us to improve our manuscript.

To the Associate editor:

*While revising your submission, please upload your figure files to the Preflight Analysis and Conversion Engine (PACE) digital diagnostic tool, https://pacev2.apexcovantage.com/. PACE helps ensure that figures meet PLOS requirements. To use PACE, you must first register as a user. Registration is free. Then, login and navigate to the UPLOAD tab, where you will find detailed instructions on how to use the tool. If you encounter any issues or have any questions when using PACE, please email PLOS at figures@plos.org. Please note that Supporting Information files do not need this step.*

Response: Thank you for the opportunity to check the figures. We used the suggested website and modified to meet *PLOS* requirements.

*Reviewer #1:*

*All comments from authors have been addressed. However, I want to ask the authors to add the details of their power analysis.*

*They write:*

*Page 5, line 114*

*A priori power analysis indicated that a sample size of at least 28 was necessary*

*Only this sentence is not enough to provide evidence of a well-performed power analysis. The authors need to report in the paper exactly how they performed this analysis.*

*For instance: type of test used, number of the group, correlation, alpha level, power and the effects size used.*

*If the effects size used to calculate the power is based on literature, the authors need to indicate the effects size used according to literature and cite those papers.*

*What kind of design did they indicate in this analysis? How much power, correlation and effect size they indicated to obtain that a sample size of 28 was big enough to support their effects?*

*All these details are missing and I fatigue again to accept the manuscript in this shape.*

Response: We apologize that our description was inadequate. We added in our manuscript that we conducted our power analysis by the following study design, *F*-test with two groups with three measurements, and parameters as alpha = .05, power = 0.80, and correlation = 0.50. Since previous studies did not describe the effect sizes in their papers, the traditional effect size of 0.25 was used in our research. We cited Cohen's criteria to determine the effect size. We also added information about the software to calculate the result.

[Added text]

Page 5, line 114-118

We performed a priori power analysis for the sample size estimation for *F*-test based on our study design, two groups with three measurements, using GPower 3.1 [38]. Assuming that this study has a medium effect size, we used 0.25 based on Cohen's criteria [39]. With an alpha = .05, power = 0.80, and correlation = 0.50, it indicated that a sample size of at least 28 was necessary.
